# The Impact of Concomitant Proton Pump Inhibitors on Immunotherapy Efficacy among Patients with Urothelial Carcinoma: A Meta-Analysis

**DOI:** 10.3390/jpm12050842

**Published:** 2022-05-20

**Authors:** Alessandro Rizzo, Matteo Santoni, Veronica Mollica, Angela Dalia Ricci, Concetta Calabrò, Antonio Cusmai, Gennaro Gadaleta-Caldarola, Gennaro Palmiotti, Francesco Massari

**Affiliations:** 1 Struttura Semplice Dipartimentale di Oncologia Medica per la Presa in Carico Globale del Paziente Oncologico “Don Tonino Bello”, Istituto di Ricerca e Cura a Carattere Scientifico (IRCCS), Istituto Tumori Giovanni Paolo II-Bari, Viale Orazio Flacco 65, 70124 Bari, Italy; a.cusmai@oncologico.bari.it (A.C.); gennaropalmiotti@hotmail.it (G.P.); 2 Oncology Unit, Macerata Hospital, 62100 Macerata, Italy; mattymo@alice.it; 3 Medical Oncology, IRCCS Azienda Ospedaliero-Universitaria di Bologna, Via Albertoni-15, 40138 Bologna, Italy; veronica.mollica7@gmail.com (V.M.); f.massari79@gmail.com (F.M.); 4 Departmental Unit of Medical Oncology, ‘San Paolo’ Hospital, ASL BA, 70123 Bari, Italy; dalia.ricci@gmail.com; 5 S.C. Farmacia e U.Ma.C.A., Istituto di Ricerca e Cura a Carattere Scientifico (IRCCS), Istituto Tumori Giovanni Paolo-Bari, 70124 Bari, Italy; concetta.calabro@oncologico.bari.it; 6 Medical Oncology Unit, ‘Mons. R. Dimiccoli’ Hospital, Azienda Sanitaria Locale Barletta, 76121 Barletta, Italy; gergad@libero.it; 7 Department of Experimental, Diagnostic and Specialty Medicine, University of Bologna, 40138 Bologna, Italy

**Keywords:** atezolizumab, durvalumab, immunotherapy, meta-analysis, nivolumab, pembrolizumab, proton pump inhibitors, urothelial carcinoma

## Abstract

Background. Immune checkpoint inhibitors (ICIs) have recently represented a breakthrough in urothelial carcinoma (UC). Proton pump inhibitors (PPIs) are routinely used for extended time periods in UC patients, with these agents having potentially and frequently undervalued effects on ICIs efficacy. Methods. We performed a meta-analysis aimed at investigating the impact of concomitant PPI administration on progression-free survival (PFS) and overall survival (OS) among patients receiving immunotherapy for metastatic UC. Results. Two studies encompassing a total of 1015 patients were included. The pooled Hazard Ratios (HRs) for OS and PFS were 1.55 (95% CI, 1.31–1.84) and 1.43 (95% CI, 1.23–1.66), respectively, suggesting that the administration of PPIs was negatively associated with PFS and with OS in UC patients treated with ICIs. Conclusions. The current meta-analysis represents the first study to provide a systematic evaluation of the impact of concomitant PPI use in UC patients treated with ICIs. Further studies are warranted on this topic to clarify the relationship between gut microbiome, antiacid exposure, and cancer immunotherapy. In the current era of medical oncology, progress in this setting will require the collaboration of basic science and clinical research to optimize systemic treatment and to improve the outcomes of UC patients receiving ICIs.

## 1. Introduction

Urothelial carcinoma (UC) represents the ninth most frequently diagnosed malignancy worldwide, with nearly half a million new diagnoses annually [1]. While most UCs (70–75%) present with non-muscle invasive disease, approximately 25–30% of patients are affected by muscle-invasive bladder cancer (MIBC) or metastatic UC at the time of diagnosis [2,3]. Following preclinical studies and clinical evidence suggesting the immunogenicity of UC, immunotherapy has been tested in several settings, ranging from neo(adjuvant) to metastatic, and the emerging of immune checkpoint inhibitors (ICIs) has recently represented a breakthrough in treatment-naïve and previously treated UC patients [4,5,6]. In fact, we have recently witnessed the approval of five ICIs in the last few years (pembrolizumab, nivolumab, atezolizumab, durvalumab, and avelumab) by the Food and Drug Administration (FDA) and the practice-changing JAVELIN Bladder 100 phase III trial has reported unprecedented clinical benefits for avelumab maintenance in UC patients without disease progression with first-line platinum-based chemotherapy [7,8]. However, a non-negligible proportion of UCs treated with ICIs do not benefit from this therapeutic approach due to several reasons, including the lack of reliable predictors of treatment response and resistance, such as PD-L1 expression, tumor mutational burden (TMB), microsatellite instability (MSI) status, and gut microbiota [9,10]. In fact, the identification of biomarkers and elements predicting as well as impairing response to ICIs remains of pivotal importance, especially considering that the number of indications and UC patients receiving ICIs is supposed to further increase soon [11,12].

Cancer patients frequently receive multiple medications due to preexisting comorbidities as well as treatment-related side effects [13,14]. Among these, proton pump inhibitors (PPIs) are routinely used for extended time periods in this patient population, with these agents having potentially and frequently undervalued effects on ICIs efficacy [15,16]. Recent studies have suggested that PPI use may cause changes in the composition of gut microbiota, also modifying the response to immunotherapy [17,18]. Conversely, other trials have shown no effects determined by concomitant PPI use among cancer patients treated with ICIs, and few data are available in genitourinary malignancies, including UC [19,20]. Thus, the question of whether concomitant medications such as PPIs could determine the reduced efficacy of ICIs remains a priority area [21,22]. In the current paper, we performed a meta-analysis aimed at investigating the impact of concomitant PPIs on progression-free survival (PFS) and overall survival (OS) among patients receiving immunotherapy for metastatic UC.

## 2. Materials and Methods

### 2.1. Search Strategies

All clinical trials published from 10 June 2000 to 30 December 2021, were searched. Keywords used for searching on PubMed/ Medline, Cochrane Library and EMBASE were: “immunotherapy” OR “nivolumab” OR “ipilimumab” OR “atezolizumab” OR “pembrolizumab” OR “durvalumab” OR “avelumab” OR “immune checkpoint inhibitors” AND “metastatic disease” AND “urothelial carcinoma” OR “bladder cancer” OR “bladder carcinoma” AND “proton pump inhibitors” OR “PPI” OR “omeprazole” OR “pantoprazole” OR “lansoprazole” OR “esomeprazole” OR “rabeprazole”. Only articles published in peer-reviewed journals, written in the English language, and with available full text were considered. Three authors evaluated the search and review of the articles independently.

### 2.2. Data Extraction and Quality Assessment

The following data were extracted for each publication: (1) study information (author, carry out country, inclusion criteria); (2) type and dose of ICI; (3) number of patients. Three authors assessed the quality of included studies according to the Newcastle–Ottawa quality assessment scale (NOS), which considered subject selection, comparability, and the evaluation of the outcome. The current analysis was conducted according to PRISMA guidelines (Supplementary Table S1) [23,24].

The PICO questions were formulated as follows:

- Population: metastatic UC patients;
- Intervention: concomitant PPIs and ICIs;
- Control: ICIs;
- Outcome: OS and PFS.

### 2.3. Statistical Design

All statistical analyses were performed using ProMeta 3 software.

Effect measures for OS were Hazard Ratios (HRs) and 95% Confidence Intervals (CIs). HRs were assessed by forest plots. The Chi-square test and the I^2^ statistic were used to examine statistical heterogeneity; substantial heterogeneity was considered to exist when the I^2^ value was greater than 50% or there was a low *p* value (<0.10) in the Chi-square test [25].

### 2.4. Primary and Secondary Endpoints

The co-primary endpoints of the meta-analysis were:

- To assess PFS in UC patients treated with concomitant PPIs and ICIs;
- To assess OS in UC patients treated with concomitant PPIs and ICIs.

## 3. Results

### 3.1. Search Results

**Figure 1:**
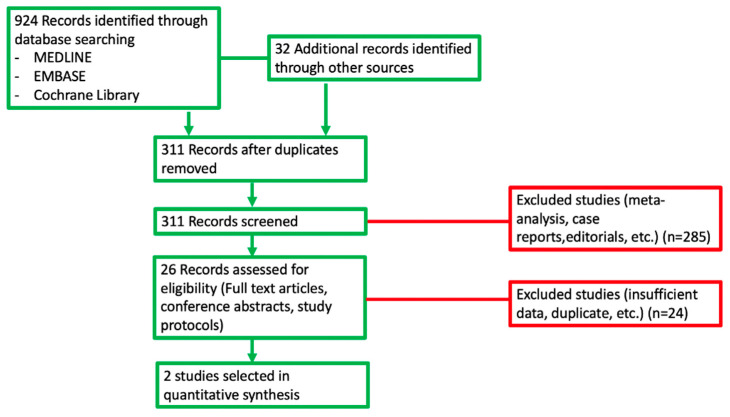
Selection of randomized controlled trials (RCTs) included in the meta-analysis according to PRISMA statement.

In our search, we found 956 potentially relevant reports, which were subsequently restricted to 2 [26,27]. We excluded 954 records as non-pertinent reports, as shown in Figure 1.

Table 1 reports a summary of the included studies [26,27]. Two studies encompassing a total of 1015 patients were included.

### 3.2. Overall Survival

**Figure 2:**
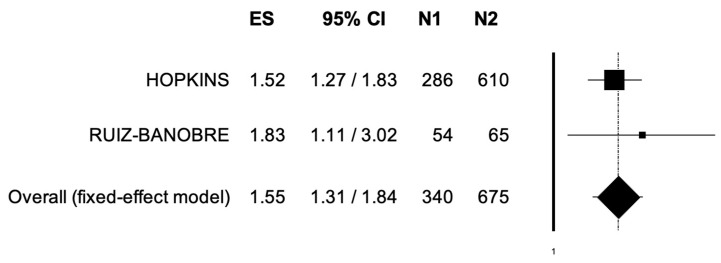
Forest plot of comparison between urothelial carcinoma patients receiving immune checkpoint inhibitors with concomitant PPIs use (N1) or not (N2); the outcome was Hazard Ratio of Overall Survival. Abbreviations: CI: confidence interval; ES: Effect Size.

The pooled HR for OS was 1.55 (95% CI, 1.31–1.84) (Figure 2), suggesting that patients receiving ICIs and PPIs presented lower OS compared to patients without concomitant PPIs administration; the analysis was associated to low heterogeneity (I^2^ of 0%), and thus a fixed-effects model was used.

### 3.3. Progression-Free Survival

**Figure 3:**
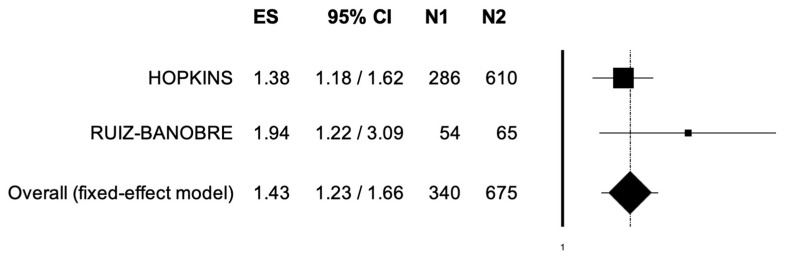
Forest plot of comparison between urothelial carcinoma patients receiving immune checkpoint inhibitors with concomitant PPIs use (N1) or not (N2); the outcome was Hazard Ratio of Progression-Free Survival. Abbreviations: CI: confidence interval; ES: Effect Size.

The pooled HR for PFS in the comparison between UC patients receiving immunotherapy with or without concomitant PPIs was 1.43 (95% CI, 1.23–1.66) (Figure 3). The analysis showed low heterogeneity, and a fixed-effect model was used (I^2^ = 0%).

## 4. Discussion

The advent of modern immunotherapy has represented a historical step forward in the management of several hematological and solid tumors, including metastatic UC [28,29]. Treatment paradigms of UC have witnessed important changes within a few years, and this rapidly changing landscape has prompted clinicians to consider the expansion of the role of ICIs to other settings, including the earlier stages of the disease [30,31]. To the best of the authors’ knowledge, the current study represents the first meta-analysis in literature to provide a systematic evaluation of the impact of concomitant PPI use in UC patients receiving immunotherapy. Our meta-analysis has highlighted inferior OS and PFS in patients treated with PPIs, and the analysis presented no heterogeneity. These findings further support the exploration of the role of concomitant medications in UC patients treated with ICIs, given the potentially meaningful clinical impact of these agents. In addition, our results suggested that the identification of specific factors (e.g., concomitant medications) modifying the response to ICIs represents an important challenge in UC; in fact, only a proportion of patients seems to benefit from immunotherapy, highlighting the need for a deeper understanding of predictors of response and resistance.

In recent years, several retrospective multicenter trials have investigated the impact of concomitant medications (e.g., metformin, aspirin, PPIs, etc.) on ICI efficacy, reporting controversial, and frequently conflicting, results [32,33]. These commonly used drugs have been suggested to negatively affect the activity of ICIs through immunomodulatory effects; in particular, agents such as PPIs may induce a detrimental effect on gut microbiota, an established and well-known regulator of immune homeostasis [34,35]. According to a recent study published by Buti and colleagues, a prognostic score based on antibiotics, PPIs, and high-dose corticosteroid therapy may be a useful tool able to stratify cancer patients receiving immunotherapy [36]. However, the question of how concomitant medications such as PPIs could enhance or decrease immunotherapy responses remains unanswered so far.

In our view, some points deserve discussion. Among these, it is more than likely that UC patients included in the two trials were taking more than one concomitant medication, and since it is not possible to fully account for these effects, this bias cannot be avoided. Due to the high prevalence of “polypharmacy” among UC patients treated with ICIs, a deeper understanding of the impact of drugs like PPIs on immunotherapy efficacy and toxicity is mandatory. At the same time, despite our belief that our results are of interest and the meta-analysis represents the first study to be specifically oriented on this topic, we are aware that available evidence is not sufficient to associate worse clinical outcomes in patients who are taking PPIs while being treated with ICIs.

From a biological point of view, antiacids such as PPIs have been reported to affect the gut microbiome through several mechanisms, including changes in gastric pH and the decrease of bacterial richness [37]; in addition, recent studies have highlighted a putative correlation between antiacid treatment and community acquired pneumococcal pneumonia, something that suggests that PPIs could affect immune system physiological function [38]. In addition, preclinical studies have reported impaired natural killer cell and neutrophil activity, which may play a role in decreasing the efficacy of ICIs in cancer patients [39,40].

Some strengths and limitations of our meta-analysis should be highlighted. Among the strengths of this study, our analysis includes an overall large number of metastatic UC patients treated with ICIs, and it represents the first study specifically focused on this important and frequently underdiscussed topic. However, some limitations should be acknowledged. First, the meta-analysis was based on aggregate data and not on individual-patient data; second, the two included trials investigated the role of different ICIs, with these studies also presenting important differences in terms of study design and patient population. Since pembrolizumab, nivolumab, atezolizumab, and durvalumab present not superimposable mechanisms of action, this element should be considered. Based on these premises, selection bias cannot be excluded. Thirdly, it was not possible to include in our analysis the impact of PPIs on toxicity. In addition, the meta-analysis was based on only two clinical trials, an important issue that should be highlighted; thus, the results of our analysis should be interpreted with caution.

The current meta-analysis suggested that PPIs administration was associated with shorter PFS and OS in UC patients treated with ICIs, corroborating the results of some post-hoc analyses and large retrospective reports suggesting the negative predictive role of PPIs use in metastatic UC patients receiving ICIs. Despite it was possible to include only two clinical trials, our study has the potential to raise awareness of this emerging topic. Other recent reports have suggested that PPIs could negatively affect the efficacy of immunotherapy through immuno-modulatory effects—for example, these agents may induce a detrimental effect on the immune system and the gut microbiome, which is known to play a key role in modifying immune homeostasis. In the current era of medical oncology, a fundamental point will be to better define how microbiota could interact with UC, and further studies are warranted on this topic to clarify the relationship between gut microbiome, antiacid exposure, and cancer immunotherapy. Progress in this setting will require the collaboration of basic science and clinical research to optimize systemic treatment and to improve the outcomes of UC patients receiving ICIs.

## Figures and Tables

**Table 1:** Summary of all the included studies in the present meta-analysis.

Author (Year)	Country	Number pts PPIs/no PPIs	Median Follow-up Time	ECOG-PS 0 or 1 (Percentage)	Median Age with Range	Type of ICIs	Newcastle–Ottawa Quality Assessment Scale
Ruiz-Banobre (2021) [27]	Spain	54/65	9.5 months	83%	69 (38–89)	Atezolizumab, Durvalumab, Nivolumab, Pembrolizumab	7
Hopkins (2020) [26]	Europe, North America, Asia—Pacific region	286/610	11 and 17 months	100%	66 (36–84) and 67 (33–88)	Atezolizumab	8

Abbreviations: ICIs: immune checkpoint inhibitors; PPIs: proton pump inhibitors; pts: patients.

## Data Availability

Not applicable.

## Supplementary Materials

The following supporting information can be downloaded at: www.mdpi.com/article/10.3390/jpm12050842/s1, Table S1: PRISMA guidelines [41].
